# Mechanism of Action of Troculeucel (Non‐genetically Modified Natural Killer Cells with Enhanced Cytotoxicity) in Alzheimer's Disease Confirmed by Corresponding Phase I Biomarker Data.

**DOI:** 10.1002/alz70859_101711

**Published:** 2025-12-25

**Authors:** Paul Y Song, Lucia Hui, Katia Betito, Sean Hong, Yoonmi Kang, Harry Chung, Jesse Carr, Cesar Alejandro Amescua, Blanca Isaura Acosta Gallo, Rufino Menchaca Díaz, Clemente Humberto Zúñiga Gil, Juan Mata, Minchan Gil, Hank Lee, Yongman Kim

**Affiliations:** ^1^ NKGen Biotech, Santa Ana, CA USA; ^2^ Behavioral Research Specialists, LLC, Glendale, CA USA; ^3^ Hospital Angeles, Tijuana, EM Mexico; ^4^ Tijuana General Hospital, Tijuana, EM Mexico

## Abstract

**Background:**

Misfolded protein deposits illicit a cascade of autoreactive T‐cell mediated neuroinflammation and damage in Alzheimer's disease (AD). These T cells have high CXCR3 expression enabling them to cross the BBB. Natural Killer (NK) cells are a key component of the innate immune system that have shown potential to slow amyloid deposition and eliminate autoreactive T‐cells and damaged neurons via NKG2D and DNAM‐1 expression. Troculeucel is a first‐in‐kind, autologous non‐genetically modified NK cell product with significant increased cytotoxicity and over 90% activating receptor expression that can be consistently produced from any donor.

**Method:**

In vitro studies were performed to evaluate whether Troculeucel could identify and eliminate amyloid and alpha‐synuclein proteins, as well as auto‐reactive T cells. Additionally, Troculeucel was administered to 13 patients in two Phase I trials (NCT04678453 and NCT06189963). The primary endpoint was safety while secondary endpoints included changes in cognitive assessment and CSF/plasma biomarkers. Troculeucel was also characterized for CXCR3 expression and other activating receptors required to help NK cells identify auto‐reactive T cells.

**Result:**

In vitro studies comparing Troculeucel to microglia (HMC3 cells) found Troculeucel could phagocytose and digest amyloid and alpha‐synuclein proteins similar to microglia. Troculeucel exhibited 99% DNAM‐1 and NKG2D expression and was only activated in the presence of autoreactive T‐cells while leaving resting T cells alone. Troculeucel was also found to have high CXCR3 expression (91.25%). Combined Phase I data revealed that 12/13 (92.3%) patients had stable or improved ADCOMS scores at three months (Week 11 for NCT04678453 and Week 14 for NCT06189963). Troculeucel crossed the BBB after simple IV administration, leading to dose‐dependent improvement in CSF levels of AB 42/40, p‐tau, and alpha‐synuclein. Notably, 9/13 (69.23%) of patients showed significant improvement in CSF levels of GFAP at three months including 100% at the highest dose level of 6 x 10^9^ cells per infusion.

**Conclusion:**

Troculeucel is consistently produced from AD patients and can cross the blood‐brain barrier via CXCR3 to improve CSF levels of amyloid and alpha‐synuclein proteins, while targeting autoreactive T cells to reduce neuroinflammation. Reduction of GFAP appears to be correlated with stabilization or improvement in ADCOMS scores observed in the study.

